# Supplementary material for: Oculomotor freezing reflects tactile temporal expectation and aids tactile perception
Source: Nat Commun. 2020 Jul 3;11:3341. doi: 10.1038/s41467-020-17160-1 (PMC7335189; doi:10.1038/s41467-020-17160-1)
Supplement: Supplementary file 3 — Reporting Summary [file 41467_2020_17160_MOESM3_ESM.pdf]

nature research

Corresponding author(s): Stephanie Badde

Last updated by author(s): May 28, 2020

Reporting Summary

Nature Research wishes to improve the reproducibility of the work that we publish. This form provides structure for consistency and transparency in reporting. For further information on Nature Research policies, see Authors & Referees and the Editorial Policy Checklist.

Statistics

For all statistical analyses, confirm that the following items are present in the figure legend, table legend, main text, or Methods section.

n/a	Confirmed	
☐	☒	The exact sample size (*n*) for each experimental group/condition, given as a discrete number and unit of measurement
☐	☒	A statement on whether measurements were taken from distinct samples or whether the same sample was measured repeatedly
☐	☒	The statistical test(s) used AND whether they are one- or two-sided<br>*Only common tests should be described solely by name; describe more complex techniques in the Methods section.*
☐	☒	A description of all covariates tested
☐	☒	A description of any assumptions or corrections, such as tests of normality and adjustment for multiple comparisons
☐	☒	A full description of the statistical parameters including central tendency (e.g. means) or other basic estimates (e.g. regression coefficient) AND variation (e.g. standard deviation) or associated estimates of uncertainty (e.g. confidence intervals)
☐	☒	For null hypothesis testing, the test statistic (e.g. *F*, *t*, *r*) with confidence intervals, effect sizes, degrees of freedom and *P* value noted<br>*Give P values as exact values whenever suitable.*
☒	☐	For Bayesian analysis, information on the choice of priors and Markov chain Monte Carlo settings
☐	☒	For hierarchical and complex designs, identification of the appropriate level for tests and full reporting of outcomes
☐	☒	Estimates of effect sizes (e.g. Cohen's *d*, Pearson's *r*), indicating how they were calculated

Our web collection on statistics for biologists contains articles on many of the points above.

Software and code

Policy information about availability of computer code

Data collection	The experimental program was written in Matlab (R2018b; The Mathworks, Natick, MA, USA) and used the Psychophysics Toolbox extension. The scripts are available at https://osf.io/7zsrq/?view_only=ad317978f6374fcc9459e1e957b5c069 [https://dx.doi.org/10.17605/OSF.IO/7ZSRQ].
Data analysis	The experimental program was written in R (R version 3.6.1; R Core Team, Vienna, Austria). The scripts are available at https://osf.io/7zsrq/?view_only=ad317978f6374fcc9459e1e957b5c069 [https://dx.doi.org/10.17605/OSF.IO/7ZSRQ].

For manuscripts utilizing custom algorithms or software that are central to the research but not yet described in published literature, software must be made available to editors/reviewers. We strongly encourage code deposition in a community repository (e.g. GitHub). See the Nature Research guidelines for submitting code & software for further information.

Data

Policy information about availability of data

All manuscripts must include a data availability statement. This statement should provide the following information, where applicable:

- Accession codes, unique identifiers, or web links for publicly available datasets
- A list of figures that have associated raw data
- A description of any restrictions on data availability

The raw data underlying all figures is available at https://osf.io/7zsrq/?view_only=ad317978f6374fcc9459e1e957b5c069 [https://dx.doi.org/10.17605/OSF.IO/7ZSRQ]..

Field-specific reporting

Please select the one below that is the best fit for your research. If you are not sure, read the appropriate sections before making your selection.

☐ Life sciences ☒ Behavioural & social sciences ☐ Ecological, evolutionary & environmental sciences

For a reference copy of the document with all sections, see nature.com/documents/nr-reporting-summary-flat.pdf

Behavioural & social sciences study design

All studies must disclose on these points even when the disclosure is negative.

Study description	Participants performed a tactile frequency discrimination task. We manipulated the temporal predictability of the tactile targets using tactile cues, which preceded the target by either constant (high predictability) or variable (low predictability) time intervals and constantly monitored participants' eye position.
Research sample	The sample consisted of members of NYU's Department of Psychology and members of the community (26 right-handed, 10 male, 19-37 years old, mean 27 years). The sample was chosen based on convenience and is representative for the scope of our study, healthy members of the general population.
Sampling strategy	Participants were recruited based on convenience sampling. The sample size was increased by 50% compared to parallel studies which in turn already tested 100% larger sample as identified based on a simulation-based power analysis.
Data collection	Participants indicated their tactile frequency percepts via a keyboard (2 alternative forced choice design); at the same time their eye position was tracked using an infrared eye tracker (Eyelink I, SR Research, Ontario, Canada). Data collection was computer-controlled through custom-written Matlab (The Mathworks, Natick, MA, USA) code which interfaced with commercial eye tracking software (SR Research, Ontario, Canada). During the experiment only the participant and the researcher were in the room; the participant was not instructed about the different conditions, the experimenter was aware of the different conditions but blind to the condition being tested at specific moments in time.
Timing	Data acquisition started 9/2019 and finished 12/2019
Data exclusions	Trials with blinks (9.6% of all trials) or saccades larger than 1 dva (4.2% of all trials) within a time interval ranging from 1000 ms before to 200 ms after the target stimulus were excluded from all eye data analyses. We pre-established to exclude these trials as blinks and larger saccades influence the occurrence of microsaccades, our main dependent measure.
Non-participation	One participant aborted the experiment because the person experienced problems fixating the center of the screen as required by our design.
Randomization	Participants were not allocated to experimental groups; every participant completed all conditions in randomized order.

Reporting for specific materials, systems and methods

We require information from authors about some types of materials, experimental systems and methods used in many studies. Here, indicate whether each material, system or method listed is relevant to your study. If you are not sure if a list item applies to your research, read the appropriate section before selecting a response.

Materials & experimental systems

n/a	Involved in the study	
☒	☐	Antibodies
☒	☐	Eukaryotic cell lines
☒	☐	Palaeontology
☒	☐	Animals and other organisms
☐	☒	Human research participants
☒	☐	Clinical data

Methods

n/a	Involved in the study	
☒	☐	ChIP-seq
☒	☐	Flow cytometry
☒	☐	MRI-based neuroimaging

Human research participants

Policy information about studies involving human research participants

Population characteristics	see Research sample
Recruitment	Participants were recruited via advertisement within NYU's Department of Psychology. Self-selection biases were reduced by offering monetary or course credit compensation. Self-selection biases are very unlikely to influence the reported results, because all results are based on comparisons within participants across conditions unknown to the participants.

Ethics oversight	The study was approved by the internal review board of New York University's Psychology Department.

Note that full information on the approval of the study protocol must also be provided in the manuscript.
