# Supplementary material for: Oculomotor freezing reflects tactile temporal expectation and aids tactile perception
Source: Nat Commun. 2020 Jul 3;11:3341. doi: 10.1038/s41467-020-17160-1 (PMC7335189; doi:10.1038/s41467-020-17160-1)
Supplement: Supplementary file 4 — Description of Additional Supplementary Files [file 41467_2020_17160_MOESM4_ESM.pdf]

Description of Additional Supplementary Files

File Name: Supplementary Data 1
Description: Statistics of task performance effects of temporal predictability.

File Name: Supplementary Data 2
Description: Statistics of microsaccade rate effects of temporal predictability across time.
